# Relative Ellipsoid Zone Reflectivity in Macular Telangiectasia Type 2

**DOI:** 10.1167/iovs.64.10.21

**Published:** 2023-07-18

**Authors:** Lukas Goerdt, Leonie Weinhold, Ben Isselmann, Jose Luis Rodriguez Garcia, Sandrine H. Künzel, Matthias Schmid, Frank G. Holz, Simone Tzaridis, Sarah Thiele

**Affiliations:** 1 Department of Ophthalmology, University of Bonn, Bonn, Germany; 2 Institute for Medical Biometry, Informatics and Epidemiology, University of Bonn, Bonn, Germany; 3 The Lowy Medical Research Institute, La Jolla, California, United States; 4 Department of Molecular Medicine, The Scripps Research Institute, La Jolla, California, United States; 5 Department of Ophthalmology, Gloucestershire Hospital NHS Foundation Trust, Cheltenham, United Kingdom

**Keywords:** macular telangiectasia type 2 (MacTel), ellipsoid zone reflectivity (EZR)

## Abstract

**Purpose:**

The relative ellipsoid zone reflectivity (rEZR) has been proposed as an innovative biomarker for photoreceptor integrity. This study evaluates the rEZR in macular telangiectasia type 2 (MacTel) eyes of different disease stages.

**Methods:**

The mean rEZR (ratio ellipsoid zone [EZ]/external limiting membrane [ELM] reflectivity [arbitrary units {AUs}], grey level range = 0-1) was analyzed for an entire spectral domain optical coherence tomography volume scan (global) and for each subfield of the Early Treatment Diabetic Retinopathy Study (ETDRS) grid (topographic) in patients with MacTel and controls. MacTel disease severity was classified according to Gass and Blodi.

**Results:**

Linear mixed-model analysis of 145 eyes of 74 patients and 50 eyes of 25 controls revealed globally lower, yet not statistically significant, rEZR values in MacTel eyes. Topographically, most pronounced decreases were found in stages 3 and 4/5 for the temporal inner (coefficient estimates [CEs] = −25.4 [−38.2; −12.6] and −34.1 [−48.7; −19.6] AU, both: *P* < 0.001), the inferior inner (−29.9 [−44.6; −15.6] and −35.3 [−52.1; −18.5] AU, both: *P* < 0.001), the nasal inner (−21.5 [−35.52; −7.4] and −31.6 [−47.6; −15.6] AU, *P* = 0,003 and *P* < 0.001), and in the superior inner subfield of stage 4/5 (−25.0 [−42.0; −7.9] AU, *P* = 0.004).

**Conclusions:**

The rEZR showed association with disease severity and the predilection area of MacTel. Given the current understanding of the pathophysiological concept of MacTel, these findings underscore the value of the rEZR as a potential novel biomarker for outer retinal integrity. Longitudinal studies are demanded to better characterize its value as a biomarker for early photoreceptor alterations and disease progression in MacTel.

Macular telangiectasia type 2 (MacTel) is a bilateral disease of the central retina, typically affecting individuals over 40 years at first onset.^1^^,^^2^ Structural alterations, for example, proliferation of intraretinal vessels and loss of the ellipsoid zone (EZ) occur initially in the temporal parafovea, which later involve an oval shaped area of 8 degrees horizontal and 5 degrees vertical diameter centered on the fovea (“MacTel area”).^2^ Disease progression also leads to photoreceptor dysfunction resulting in loss of retinal sensitivity and finally visual acuity.^3^^–^^5^

In spectral-domain optical coherence tomography (SD-OCT), photoreceptor loss in MacTel is detectable as a slowly progressive disruption of the second hyper-reflective band in the outer retina, termed the EZ.^2^ Although its quantification has become an accepted outcome measure in ongoing phase III trials (NCT trial number NCT03319849 and NCT03316300), biomarkers and treatment opportunities targeting already earlier in the disease course would be desirable. Herein, identification of high-risk eyes and/or macular regions prior to the occurrence of irreversible photoreceptor alterations and functional impairment would be needed, but reliable biomarkers are still missing.^6^^–^^11^

Given the fact that retinal visualization by SD-OCT imaging is based on refractive tissue properties, photoreceptors’ mitochondria are currently assumed to represent the source of the EZ's signal. In addition, functional complexes between Müller cells and retinal photoreceptors become apparent in SD-OCT imaging as the first hyper-reflective band and is named the external limiting membrane (ELM). The ratio of the EZ to the ELM reflectivity, termed the relative EZ reflectivity (rEZR), has recently been proposed as an auspicious biomarker for outer retinal integrity and photoreceptor health.^9^^,^^12^^–^^14^ First studies revealed an association of the rEZR with disease severity in age-related macular degeneration (AMD).^15^^,^^16^ In MacTel, however, only the pure (and uncorrected) EZ reflectivity has been assessed by Barthelmes et al. showing reduced reflectivity values in the parafoveal region.^17^ Although the study by Barthelmes and colleagues was performed in only 14 patients with MacTel, the results warrant a refined characterization of reflectivity changes in the outer retina in eyes with MacTel.

The purpose of this study is to assess the rEZR within the pathophysiological concept of MacTel with regard to disease staging and its spatial predilection of structural alterations in the retina as well as to evaluate the rEZR in MacTel eyes without manifest EZ loss.^2^ The evaluation of the rEZR within the pathophysiological concept of MacTel will be of informative value regarding its usefulness as a biomarker for outer retina integrity and photoreceptor degeneration.

## Materials and Methods

For this cross-sectional case-control prospective study, patients with MacTel and controls were selected from a single center cohort at the Department of Ophthalmology, University of Bonn, Germany. Patients were included between October 2016 and November 2022 in the context of the Natural History and Observational Registry study (NHOR; www.mactelresearch.org), a multinational natural history study on MacTel. Inclusion criteria were a confirmed MacTel diagnosis and retinal imaging of sufficient quality. Exclusion criteria included the presence of confounding ocular conditions, such as AMD and diabetic retinopathy, as well as optic media opacities. Controls were identified after a comprehensive ophthalmic examination. If both eyes of one participant fulfilled the inclusion criteria, both eyes were included. This study was approved by the local ethics committee at the University Hospital Bonn (Bonn, Germany) and adhered to the tenets of the Declaration of Helsinki and written informed consent was obtained prior to data acquisition.

All participants underwent a comprehensive ophthalmologic examination, including the assessment of best-corrected visual acuity (BCVA), a slit-lamp and dilated fundus examination, and retinal imaging. Following pupil dilatation with tropicamide 0.5% and phenylephrine 2.5% eye drops, retinal imaging was performed including 55 degrees color fundus photography (CFP; Zeiss Visucam 500; Carl Zeiss Meditec, Dublin, CA, USA), combined confocal scanning laser ophthalmoscopy (cSLO; near-infrared reflectance [IR] 30 degrees, automated real-time mode [ART] at least 30 single frames) and SD-OCT (30 degrees × 25 degrees, 121 B-scans, high speed mode; Spectralis HRA2+OCT; Heidelberg Engineering, Heidelberg, Germany). The 30 degrees × 25 degrees grid was used to allow for rEZR assessment within and beyond the MacTel area.

### Image Preprocessing and Grading

**Figure 1.**
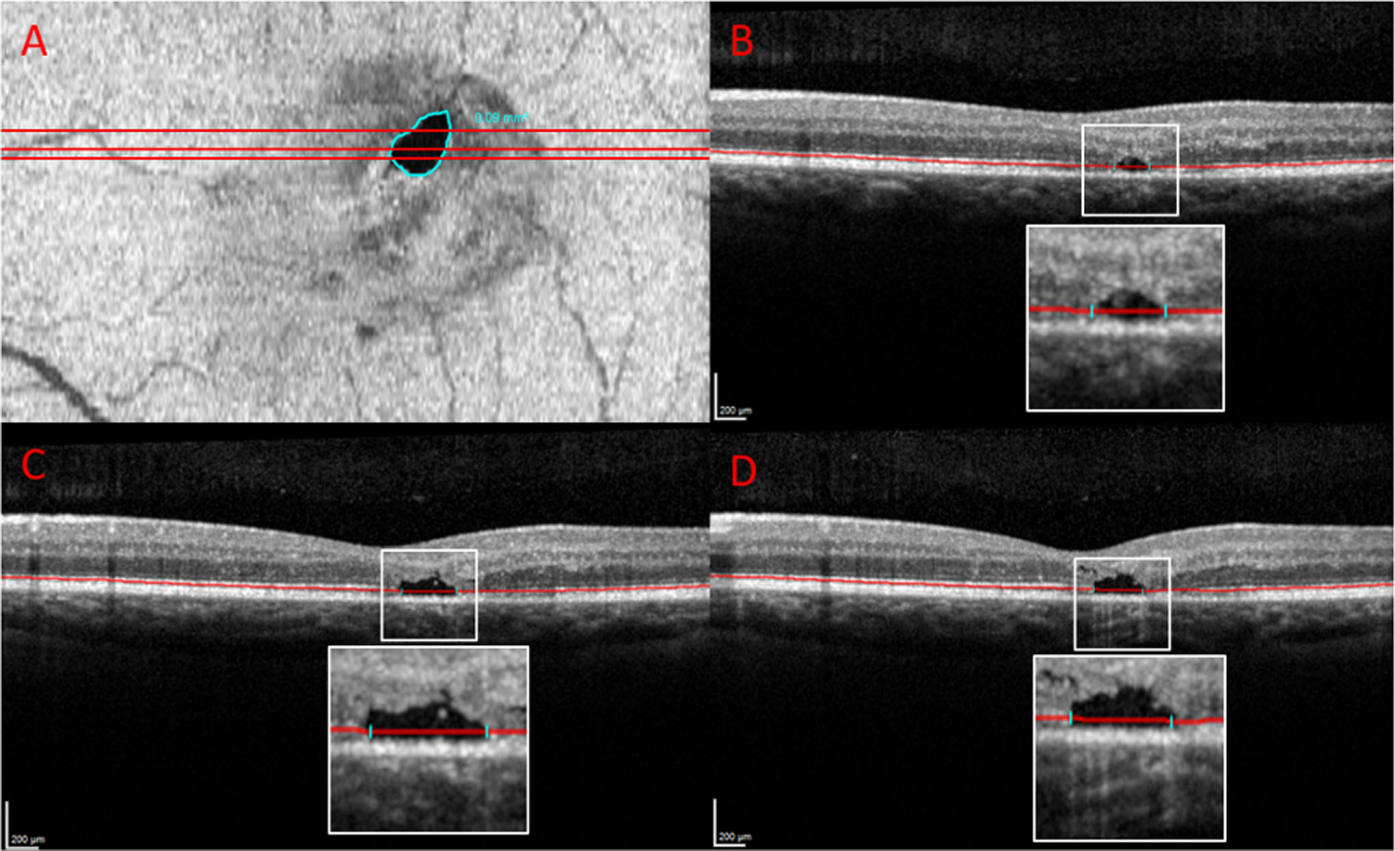
Exemplary case of ellipsoid zone (EZ) loss identification and delineation in a patient with macular telangiectasia type 2 (MacTel; left eye) with (**A**) representing en face optical coherence tomography (OCT) volume scanning. The *red lines* indicate the position of single OCT B-scans **B–D** with **B** being the upper B-scan, **C** the middle, and **D** the lower B-scan, respectively. The area of manifest EZ loss is delineated in *light blue* in the en face image (**A**) which is based on the exact alignment of the manifest EZ loss boundaries (*light blue lines*) in each of the OCT B-scans.

MacTel disease stages were classified according to Gass and Blodi by CFP assessment.^18^ Gradings were performed by two masked readers (authors L.G. and S.K.). In case of disagreement, a third reader (author S.T.) was involved to arbitrate. Manifest EZ loss was determined in SD-OCT B-scans and defined as the complete absence of the EZ. Mere attenuations of the signal with the EZ still being present were not judged as manifest EZ loss.^9^ Delineation of retinal areas with manifest EZ loss was performed in the transverse SD-OCT image presentation of the built-in Heidelberg Eye Explorer software (version 2.5.5; Heidelberg Engineering) generating en face image slabs.^10^ The so-called “photoreceptor 1” line, overlaying the EZ, was used, and manually corrected if needed, as the reference and the distance to a second line was set to 0, hence the thickness of the line was set to 1 pixel. Areas of EZ loss were delineated using the “draw region” tool and, if indicated, its boundaries adjusted after re-assessment in the traditional B-scan view. Figure 1 depicts an exemplary case for the described method. Any area of EZ loss was excluded from further statistical analysis.

### Determination of the rEZR

The rEZR, defined as the EZ/ELM reflectivity ratio, was determined, as previously described, in reflectivity profiles in the raw SD-OCT image files using a semi-automated approach using MatLab (version 9.5; The MathWorks, Natick, MA, USA; annotated code available at: https://github.com/bisselma/relEZIquantification).^19^ The ELM's reflectivity has been postulated to be stable across a wide eccentricity, to be present in the fovea, and to be one of the retinal layers undergoing least reflectivity alterations with age in most diseases.^15^^,^^20^ Although Müller cell alterations are known to occur in pathophysiological process of MacTel, these are described to be limited mainly to areas with already existing EZ loss.^4^^,^^21^ As these areas were systematically excluded from statistical analysis in the here presented study, the ELM was maintained as the reference for the rEZR calculation.

**Figure 2.**
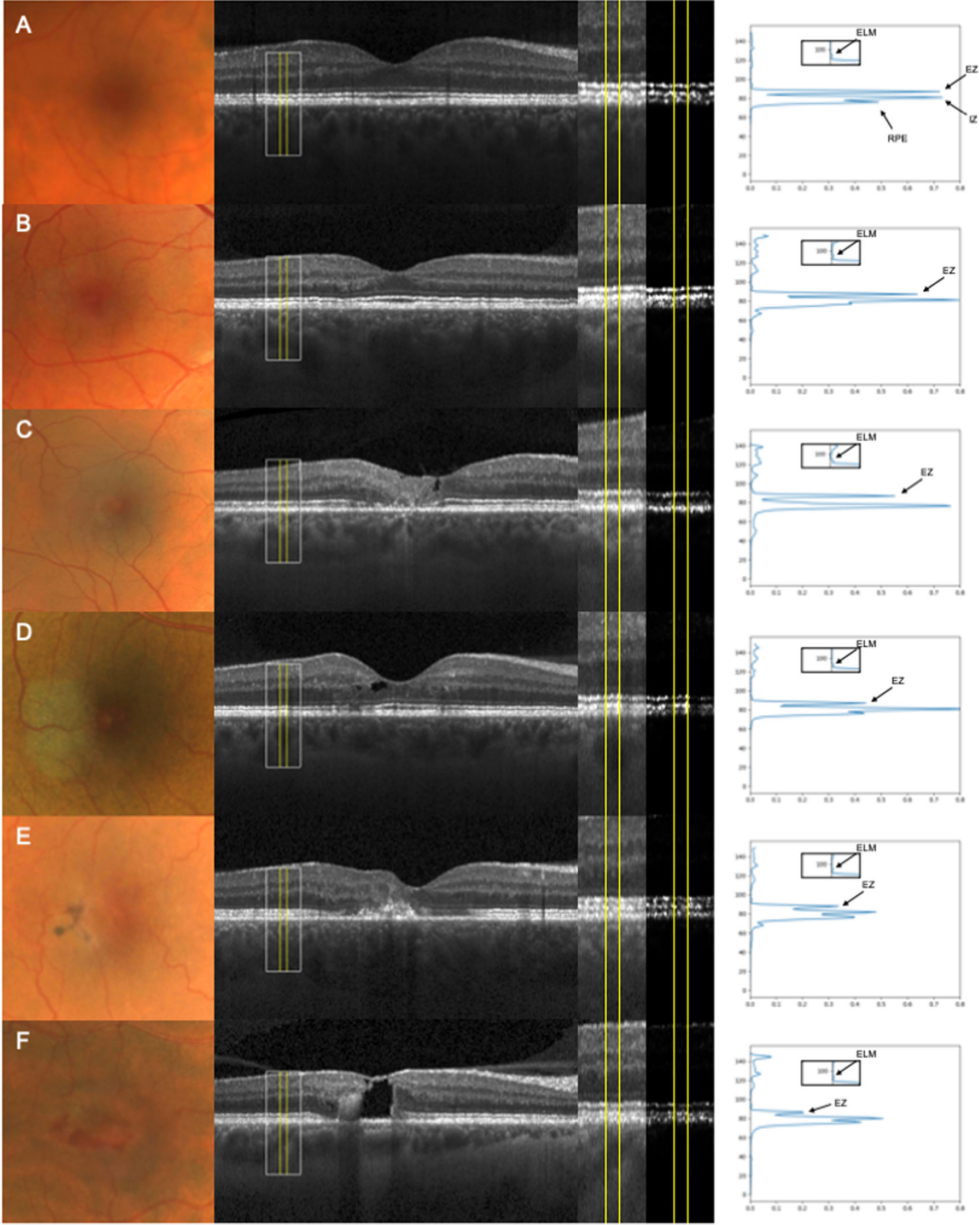
Exemplary cases for an eye of the (**A**) control group as well as of macular telangiectasia type 2 (MacTel) study group being classified as (**B**) stage 1, (**C**) stage 2, (**D**) stage 3, (**E**) stage 4, and (**F**) stage 5 demonstrated (from left to right) by color fundus photography (CFP) and the foveal spectral domain optical coherence tomography (SD-OCT) line scan with a superimposed region of interest (ROI; *yellow lines* within the *white rectangle*) for exemplary determination of the relative ellipsoid zone reflectivity (rEZR). Further, each ROI is presented magnified for the logarithmic and linear displayed SD-OCT image. Corresponding reflectivity profiles for each ROI show the specific peaks for the external limiting membrane (ELM; also as the magnified picture), ellipsoid zone (EZ), interdigitation zone (IZ), and the retinal pigment epithelium (RPE). Please note, the overall outcome of this study is the mean rEZR, which was determined as the ratio of the EZ to ELM reflectivity for the entire volume scan (global assessment) and for each of the Early Treatment Diabetic Retinopathy Study (ETDRS) subfields (topographical assessment). For the purpose of better representation, only a single reflectivity profile of a representative ROI is here presented for controls (**A**) and each MacTel disease stage (**B–F**).

Further, automated retinal layer segmentation was performed, and, if required, manually corrected using the Heidelberg Eye Explorer software (HEYEX, software version 1.10.4.0; Heidelberg Engineering). Segmentation coordinates, exported as XML files, were superimposed to the OCT raw images (i.e. native, non-logarithmic transformed data) and used for straightening of each OCT B-scan along with the coordinates of the Bruch's membrane enabling accurate rEZR determination even in eyes with pronounced posterior pole curvature. Within every single OCT B-scan, the rEZR data was obtained at adjoining regions of interests (ROIs) in corresponding reflectivity profiles (dynamic range of grey values: 0-1 [arbitrary units {AUs}]), see Figure 2A. The width of each ROI was set at 10 pixels (approximately 120  µm in high-speed SD-OCT imaging) along the image x-axis.

### Global and Topographic rEZR Assessment

**Figure 3.**
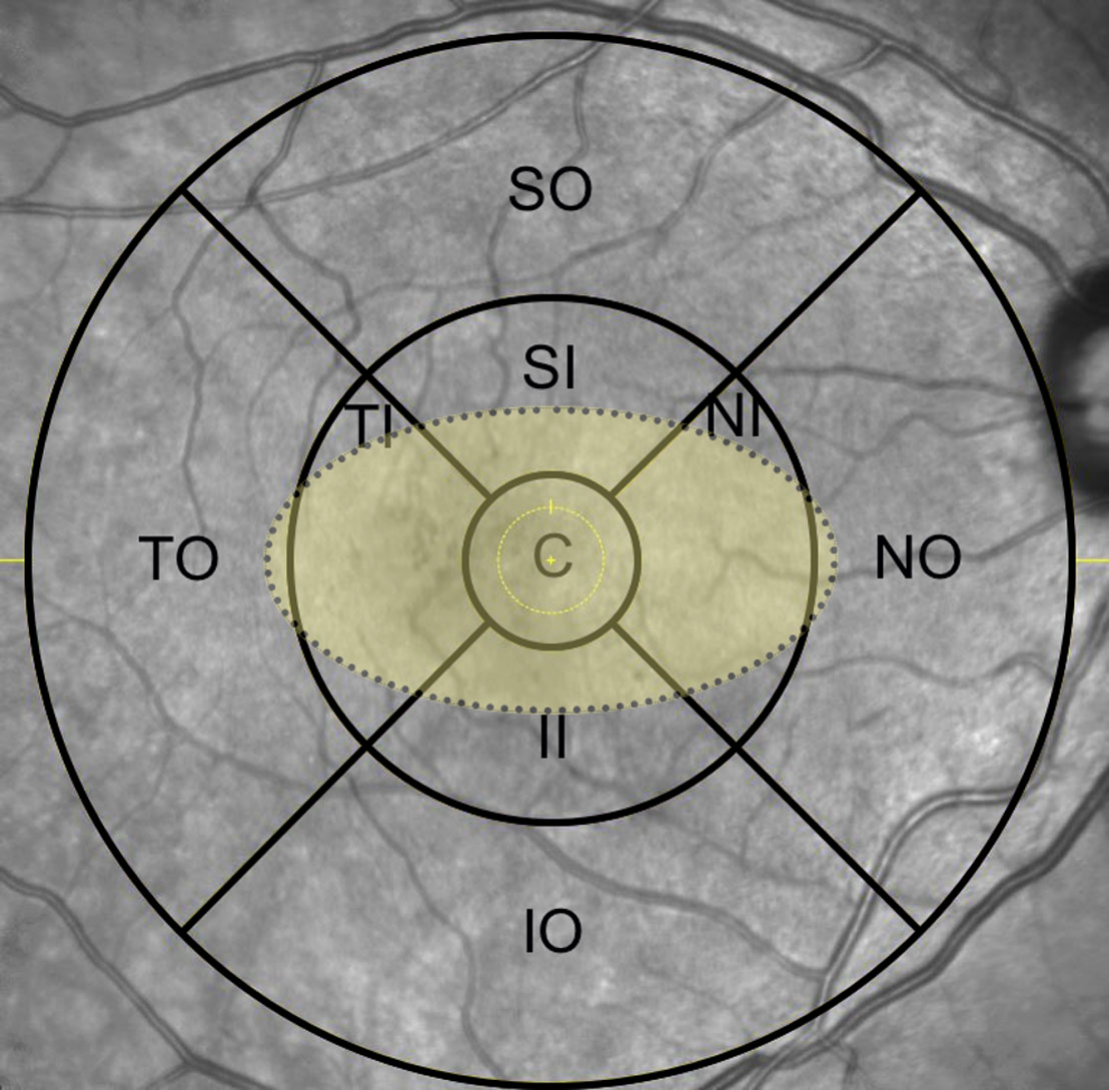
Exemplary en face near infrared (NIR) imaging of an eye with macular telangiectasia type 2 (MacTel) and the superimposed Early Treatment Diabetic Retinopathy Study (ETDRS) grid of 6 mm diameter. ETDRS subfields are as following: C = central subfield, SI = superior inner subfield, TI = temporal inner subfield, II = inferior inner subfield, NI = nasal inner subfield, SO = superior outer subfield, TO = temporal outer Subfield, IO = inferior outer subfield, IO = inferior outer subfield, and NO = nasal outer subfield. Furthermore, the so-called ellipsoid shaped “MacTel area” is highlighted with a *dotted line* and in *light yellow*.

To assess the rEZR both globally (i.e. within the entire SD-OCT volume scan) and topographically (i.e. focusing the “MacTel area”), the Early Treatment Diabetic Retinopathy Study (ETDRS) grid was used to determine the mean rEZR within each of the nine ETDRS subfields.^22^ Figure 3 gives a detailed representation of the position of the “MacTel area” within the ETDRS grid.

### Statistical Analysis

Patient characteristics are presented as absolute and relative frequencies for categorical variables and as means with standard deviations (SDs) for continuous variables. To investigate the primary research question of differences (after exclusion of retinal areas with manifest EZ loss) between rEZR values in MacTel and control eyes, linear mixed-effects models were used. These accounted for possible correlations between measurements taken from the same patient and/or eye (nested data structure with multiple data points within one eye, and 2 eyes per patient). Differences in the mean rEZR (arbitrary unit [AU]) between MacTel and control eyes were assessed (i) for the total SD-OCT volume raster scan (global assessment) and (ii) for the individual subfields (topographic assessment) of the ETDRS grid. Additionally, in eyes with MacTel, we considered retinal disease stages according to Gass and Blodi to investigate the association between MacTel disease severity and rEZR. Linear mixed-effect models were adjusted for age and eccentricity, entering the model as a spline (B-spline of degree 2). The *P* values <0.005 were considered significant (due to adjustments for multiple testing by Bonferroni-correction for 10 subfields). In order to better understand the cohort's data structure, a refined analysis of the study cohort was further performed and aimed in evaluation of the inter-eye correlation of cases and controls with bilateral inclusion as well as of the heterogeneity of the data set. The inter-eye correlation was assessed in determining Spearman's correlation coefficient *r,* which was calculated for the mean rEZR of the total SD-OCT volume raster scan of each participant with both eyes included. Further, heterogeneity of the data was evaluating by comparing the variance as well as the range of the rEZR values between patients with MacTel and controls.

For a refined characterization of the rEZR as a potential biomarker, we conducted an exploratory subgroup analysis of MacTel eyes which did not show manifest EZ loss. Here, *P* values <0.05 were considered significant. The analyses were performed with the software R, version 4.1.2, using the package lme4.^23^

## Results

### Cohort Characteristics

A total of 195 eyes of 99 study participants (mean age = 53.9 ± 12.5 years, 58 female participants) were included. Out of those, 145 eyes of 74 patients (57.2 ± 10.2 years, 41 female patients) were diagnosed with MacTel, whereas 50 eyes of 25 participants (44.1 ± 13.8 years, 17 female participants) were healthy.

MacTel staging resulted in 9 eyes (6.2%) of 9 patients with MacTel being classified as stage 1, there were 49 eyes (33.8%) of 33 patients as stage 2, there were 59 eyes (40.7%) of 43 patients as stage 3, there were 25 eyes (17.2%) of 20 patients as stage 4, and 3 eyes (2.1%) of 3 patients as stage 5, respectively. A high level of inter-reader agreement was reached, as readers 1 and 2 agreed in 140 of 145 eyes of 96.5% and a third reader was only required to arbitrate in the remaining 5 cases (3.5%). Out of the 145 MacTel eyes, manifest EZ loss was detectable in 102 eyes (70.3%) of 63 patients. In detail, 4 of the 9 stage 1 eyes demonstrated manifest EZ loss (44.4%, 4 patients), 29 of 49 stage 2 eyes (59.2%, 21 patients), 41 of 59 stage 3 eyes (69.5%, 36 patients), and 25 of 25 stage 4 eyes (100%, 20 patients), respectively. The 3 eyes classified as stage 5 also showed manifest EZ loss.

Descriptive analysis revealed a median (first quartile and third quartile) rEZR per SD-OCT volume scan of 36.9 (11.6 and 79.2) AU in MacTel eyes and of 56.9 (17.7 and 111.1) AU in control eyes. Regarding the refined analysis of the cohort's data structure, Spearman's correlation coefficient was assessed for the mean rEZR per volume raster scan in all 25 included controls and in 71 out of 75 patients with MacTel. It was revealed to be *r* = 0.164 for controls and *r* = 0.134 for patients with MacTel indicating a minor inter-eye correlation. The variance of rEZR values for MacTel eyes was more than five times higher than that of controls (24.15 vs. 135.61 AU). Consistent with these findings, the range of rEZR values in MacTel eyes was wider (on average 9.14 AU) than in controls (on average 8.40 AU). Accordingly, MacTel eyes tend to have a higher degree of heterogeneity and more extreme values than controls. Bilateral data were available from every control patient and from 71 out of 74 patients with MacTel.

Given the inhomogeneous sample size across MacTel disease stages, results of MacTel diseases stage 1 and stage 2 will be reported in the following as one group (MacTel stages 1/2) as well as of stage 4 and stage 5 (MacTel stages 4/5). Across MacTel disease stages, the median rEZR per SD-OCT volume scan was revealed to be 39.5 (15.4 and 82.9) AU in MacTel stages 1/2 eyes (*n* = 58, 36 patients), 32.1 (7.6 and 70.7) AU in stage 3 eyes (*n* = 59, 43 patients) and 28.5 (8.4 and 60.1) AU in MacTel stages 4/5 eyes (*n* = 28, 23 patients). Table 1 gives an overview of the cohort characteristics.

**Table 1.** Cohort Characteristics

	Patients With MacTel *n* = 74	Controls *n* = 25
Age, y		
Mean ± SD	57.2 ± 10.2	44.1 ± 13.8
Median [IQR]	58.20 [51.87 to 63.82]	47.29 [31.74 to 50.90]
Gender [female]		
*N* (%)	41 (55.4)	17 (68.0)
	MacTel eyes	Control eyes
	*n* = 145	*n* = 50
rEZR, AU		
Median [IQR]	36.9 [11.6 to 79.2]	56.9 [17.7 to 111.1]
Diseases stages 1/2	58 (40.0%)	
rEZR, AU, median [IQR]	39.5 [15.4 to 82.9]	
Diseases stage 3	59 (40.7%)	
rEZR, AU, median [IQR]	32.1 [7.6 to 70.7]	
Diseases stages 4/5	28 (19.3%)	
rEZR, AU, median [IQR]	28.5 [8.4 to 60.1]	

Cohort characteristics for macular telangiectasia type 2 (MacTel) and control eyes. Information is given on age (mean ± standard deviation [SD] and median with interquartile range [IQR]), gender and relative ellipsoid zone reflectivity (rEZR; arbitrary units [AUs]).

### Global rEZR Assessment Across MacTel Disease Stages

Linear-mixed models, accounting for patients’ age and the topographic dependence of the 36,626 ± 9109 (mean ± SD) rEZR data points within the SD-OCT raster scan, were applied for refined analysis of the rEZR across different MacTel disease stages. Compared to controls, global assessment revealed a decreased rEZR of all MacTel eyes (indicated by a coefficient estimate [CE] of −7.7 [−17.8 and 2.5] AU, *P* = 0.141). Considering MacTel disease staging, this rEZR decrease was more pronounced, as MacTel severity increased (CEs of −1.4 [−12.1 and 9.4] AU, *P* = 0.803, in MacTel stages 1/2, of −11.3 [−21.7 and −0.8] AU, *P* = 0.035 in stage 3 and of −11.8 [−23.5 and 0.01] AU in stages 4/5, *P* = 0.050). Figure 2 shows exemplary reflectivity profiles for distinct ROIs for each disease stage.

### Topographic rEZR Assessment

The rEZR decrease of MacTel eyes showed a topographic dependency being more pronounced in the direct parafovea compared to the foveal or more eccentric retinal region. Biggest rEZR differences between controls and MacTel eyes across all disease stages were found in the temporal inner (*P* < 0.001), inferior inner (*P* < 0.001), and nasal inner (*P* = 0.004) ETDRS subfield with CEs of −23.2 (−35.6 and −10.8) AU, −25.2 (−39.4 and −11.1) AU and −19.7 (−33.1 and −6.3) AU, respectively. Further topographic results are given in Table 2.

**Table 2.** Linear Mixed Model Results for Global and Topographic Relative Ellipsoid Zone Reflectivity (rEZR [Arbitrary Units, AUs]) Analysis of the Total MacTel Study Cohort (Reference: Control Group)

	Coefficient Estimates [AU]	95% Confidence Interval [AU]	*P* Value
Global			
Intercept:	95.8	78.2–113.5	0.141
Case:	−7.7	−17.8 to 2.5	
Center			
Intercept:	51.0	34.9–67.2	0.140
Case:	−6.6	−15.4 to 2.2	
Temporal inner			
Intercept:	105.1	83.4–126.8	**<0.001**
Case:	−23.2	−36.7 to −10.8	
Inferior inner			
Intercept:	111.7	87.1–136.4	**<0.001**
Case:	−25.2	−39.4 to −11.1	
Nasal inner			
Intercept:	95.1	71.9–118.4	**0.004**
Case:	−19.7	−33.1 to −6.3	
Superior inner			
Intercept:	113.0	87.6–138.5	0.061
Case:	−14.0	−28.6 to 0.6	
Temporal outer			
Intercept:	129.9	103.2–156.7	0.266
Case:	−8.7	−24.1 to 6.7	
Inferior outer			
Intercept:	125.6	98.7–152.6	0.152
Case:	−11.3	−26.7 to 4.1	
Nasal outer			
Intercept:	134.6	105.8–163.4	0.039
Case:	−17.4	−34.0 to −0.9	
Superior outer			
Intercept:	125.1	97.2–153.1	0.279
Case:	−8.9	−25.0 to 7.2	

Values given in arbitrary units [AUs].

**Figure 4.**
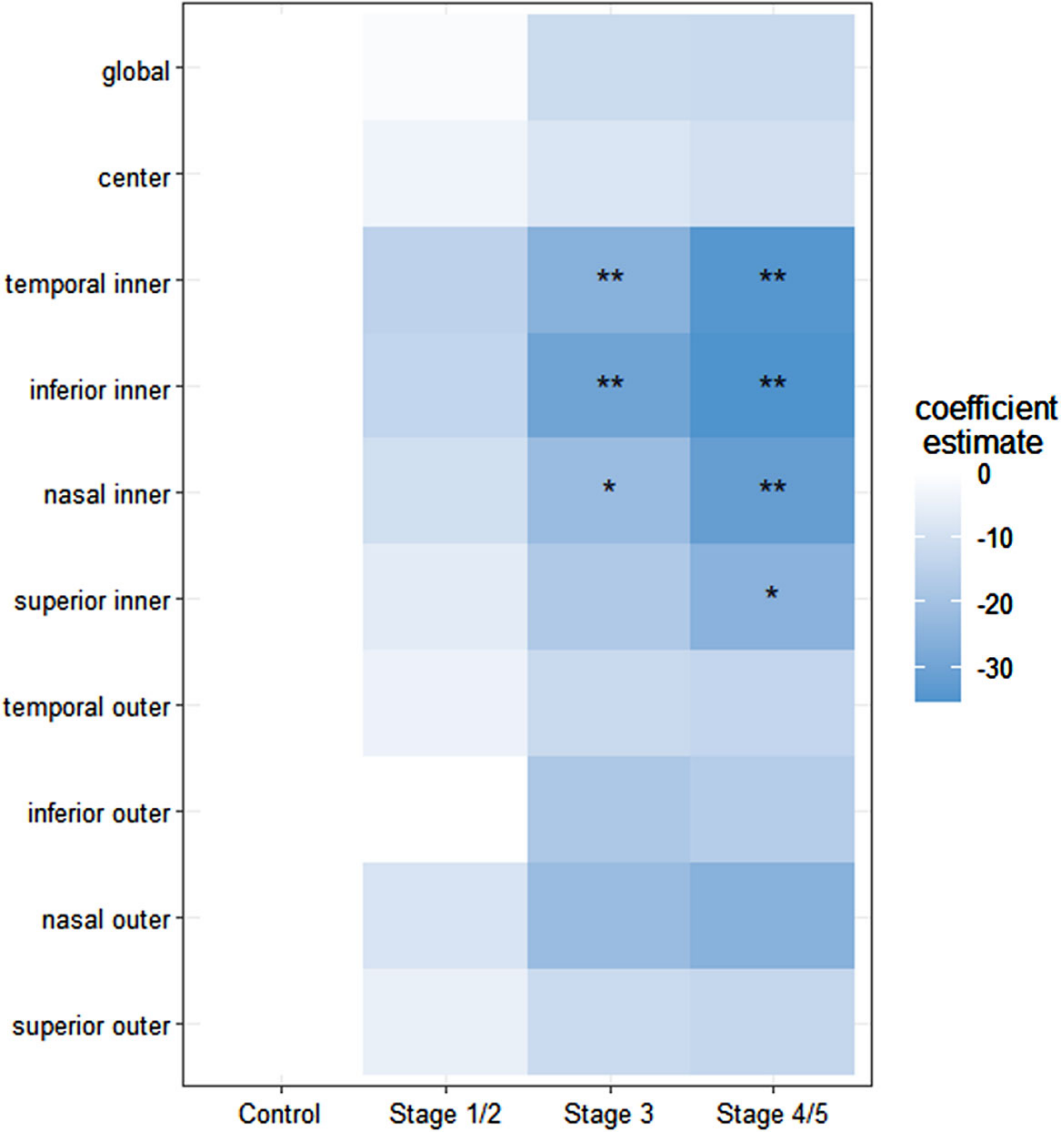
Graphical representation of linear-mixed model results for the global (*first row*) and the topographic (*second to final row*) assessment of the relative ellipsoid zone reflectivity (rEZR) and its association with macular telangiectasia type 2 (MacTel) disease staging for the total study cohort. The extent of rEZR differences between MacTel stages and healthy controls is indicated by the color-coded (from *white* *to dark blue*) coefficient estimates. Coefficient estimates of higher negative values, indicating more pronounced rEZR decreases of MacTel eyes compared to controls, are represented by darker blue colors. *P* values (** = *P* value 0.001; * = *P* value <0.005).

The associations of MacTel disease staging and the retinal region with the rEZR remained apparent when considering both simultaneously. The mean rEZR of each ETDRS subfield was shown to be more decreased with advancing disease stages and this decrease, again, was more pronounced in the pericentral ETDRS subfields. Compared to healthy eyes, the rEZR in the temporal inner subfield was decreased with a CE of −14.1 (−27.2 and −1.0) AU (*P* = 0.035) in MacTel stages 1/2 eyes, of −25.4 (−38.2 and −12.6) AU (*P* < 0.001) in stage 3 and of −34.1 (−48.7 and −19.6) AU (*P* < 0.001) in stages 4/5, respectively. In the inferior inner subfield, the rEZR was decreased with a CE of −13.2 (−28.3 and 1.9) AU (*P* = 0.087) in stages 1/2 MacTel eyes, of −29.9 (−44.6 and −15.1) AU (*P* < 0.001) in stage 3 eyes and of −35.3 (−52.1 and −18.5) AU (*P* < 0.001) in stages 4/5 eyes compared to healthy eyes. Analyses of the nasal inner and the superior inner subfield also showed decreased rEZR values with a CE of −10.0 (−24.5 and 4.5) AU (*P* = 0.177) and −6.1 (−21.5 and 9.3) AU (*P* = 0.436) in stages 1/2 eyes, of −21.5 (−35.2 and −7.4) AU (*P* = 0.003) and −17.2 (−32.3 and −2.1) AU (*P* = 0.025) in stage 3 eyes as well as of −31.6 (−47.6 and −15.6) AU (*P* < 0.001) and −25.0 (−42.0 and −7.9) AU (*P* = 0.004) in MacTel eyes classified as stages 4/5. Detailed results of the impact of MacTel disease staging and retinal topography on the rEZR are presented in Table 3. For a graphical presentation of the results, see Figure 4.

**Table 3.** Linear Mixed Model Results of Global and Topographic Relative Ellipsoid Zone Reflectivity (rEZR) Analysis Across Macular Telangiectasia Type 2 (MacTel) Disease Stages (Reference: Control Group)

	Disease Stage	Coefficient Estimates [AU]	95% Confidence Interval [AU]	*P* Value
Global	Intercept	95.7	78.5–112.9	
	1/2	−1.37	−12.1 to 9.4	0.803
	3	−11.3	−21.7 to −0.8	0.035
	4/5	−11.8	−23.5 to 0.01	0.050
Center	Intercept	50.7	34.4 to 66.9	
	1/2	−3.6	−13.6 to 6.4	0.484
	3	−8.0	−17.6 to 1.6	0.296
	4/5	−9.8	−21.2 to 1.6	0.291
Temporal inner	Intercept	103.9	83.0 to 124.8	
	1/2	−14.1	−27.2 to −1.0	0.035
	3	−25.4	−38.2 to −12.6	**<0.001**
	4/5	−34.1	−48.7 to −19.6	**<0.001**
Inferior inner	Intercept	110.6	86.6 to 134.5	
	1/2	−13.2	−28.3 to 1.9	0.087
	3	−29.9	−44.6 to −15.1	**<0.001**
	4/5	−35.3	−52.1 to −18.5	**<0.001**
Nasal inner	Intercept	94.5	71.6 to 117.4	
	1/2	−10	−24.5 to 4.5	0.177
	3	−21.5	−35.52 to −7.4	**0.003**
	4/5	−31.6	−47.6 to −15.6	**<0.001**
Superior inner	Intercept	111.3	86.4 to 136.2	
	1/2	−6.1	−21.5 to 9.3	0.436
	3	−17.2	−32.3 to −2.1	0.025
	4/5	−25	−42.0 to −7.9	**0.004**
Temporal outer	Intercept	129.4	102.9 to 156.0	
	1/2	−3.9	−20.4 to 12.5	0.639
	3	−11.8	−28.0 to 4.3	0.151
	4/5	−13.1	−31.1 to 4.8	0.151
Inferior outer	Intercept	129.4	99.5 to 152.6	
	1/2	−0.1	−17.4 to 17.2	0.993
	3	−17.8	−34.5 to −1.1	0.037
	4/5	−15.9	−35.4 to 3.6	0.109
Nasal outer	Intercept	134	105.2 to 162.7	
	1/2	−8.6	−26.8 to 9.6	0.353
	3	−21.8	−39.4 to −4.1	0.015
	4/5	−25.4	−45.1 to −5.6	0.012
Superior outer	Intercept	124.8	97.1 to 152.5	
	1/2	−4.7	−21.7 to 12.4	0.59
	3	−11.4	−28.1 to 5.3	0.182
	4/5	−12.7	−31.2 to 5.9	0.182

Values given in arbitrary units [AUs].

### Subgroup Analysis: rEZR in MacTel Eyes Without Manifest EZ Loss

Out of the total MacTel cohort, 29.7% (43 eyes/33 patients; 54.5 ± 9.2 years) showed no signs of manifest EZ loss. Five eyes (11.6%, 5 patients) were previously classified as MacTel stage 1, 20 eyes (46.5%, 18 patients) as stage 2, and 18 eyes (41.9%, 14 patients) as stage 3. Descriptive analysis showed a median rEZR of 29.5 (9.0 and 65.7) AU in MacTel eyes without EZ loss.

Linear-mixed models revealed that all included MacTel eyes without manifest EZ loss exhibited a decreased rEZR with a CE of −5.2 (−17.6 and −7.2) AU (*P* = 0.412) in the global SD-OCT assessment. Topographic analysis showed most pronounced rEZR decreases in the temporal inner (CE of −13.3 [−28.3 and 1.7] AU, *P* = 0.082) and in the inferior inner (CE of −14.6 [−32.0 and 2.8] AU, *P* = 0.101) ETDRS subfield. Table 4 summarizes the topographical results across the total MacTel subgroup without EZ loss in detail.

**Table 4.** Linear-Mixed Model Relative Ellipsoid Zone Reflectivity (rEZR) Results of all Assessed Macular Telangiectasia Type 2 (MacTel) Eyes Without Manifest Ellipsoid Zone (EZ) Loss Compared to Controls (Reference)

	Coefficient Estimates [AU]	95%-Confidence Interval [AU]	*P* Value
Global			
Intercept:	106.5	82.9 to 130.0	0.412
Case:	−5.2	−17.6 to 7.2	
Center			
Intercept:	44.9	25.1 to 64.7	0.599
Case:	2.8	−7.6 to 13.2	
Temporal inner			
Intercept:	106.5	84.6 to 141.3	0.082
Case:	−5.2	−28.3 to 1.7	
Inferior inner			
Intercept:	122.7	89.9 to 155.6	0.101
Case:	−14.6	−32.0 to 2.8	
Nasal inner			
Intercept:	96.3	64.0 to 128.6	0.417
Case:	−7.1	−24.1 to 10.0	
Superior inner			
Intercept:	125.7	91.1 to 160.3	0.397
Case:	−7.8	−25.8 to 10.2	
Temporal outer			
Intercept:	137.7	103.1 to 172.2	0.415
Case:	−7.6	−25.8 to 10.6	
Inferior outer			
Intercept:	135.9	102.3 to 169.6	0.566
Case:	−5.2	−23.1 to 12.7	
Nasal outer			
Intercept:	147.8	108.7 to 186.8	0.312
Case:	−10.8	−31.6 to 10.1	
Superior outer			
Intercept:	128.4	90.9 to 165.8	0.457
Case:	−7.4	−27.0 to 12.2	

Values given in arbitrary units [AU].

With regard to disease staging of the included MacTel eyes without manifest EZ loss, topographic analysis revealed the rEZR decrease to be more pronounced with advancing disease staging in all of the four inner ETDRS subfields. Herein, assessed stage 3 MacTel eyes without manifest EZ loss, exhibited a statistically significant rEZR decrease in the temporal inner and the inferior inner ETDRS subfield indicating CEs of −21.2 (−39.3 and −3.2) AU (*P* = 0.021) and −28.4 (−48.7 and −8.1) AU (*P* = 0.006), respectively.

**Figure 5.**
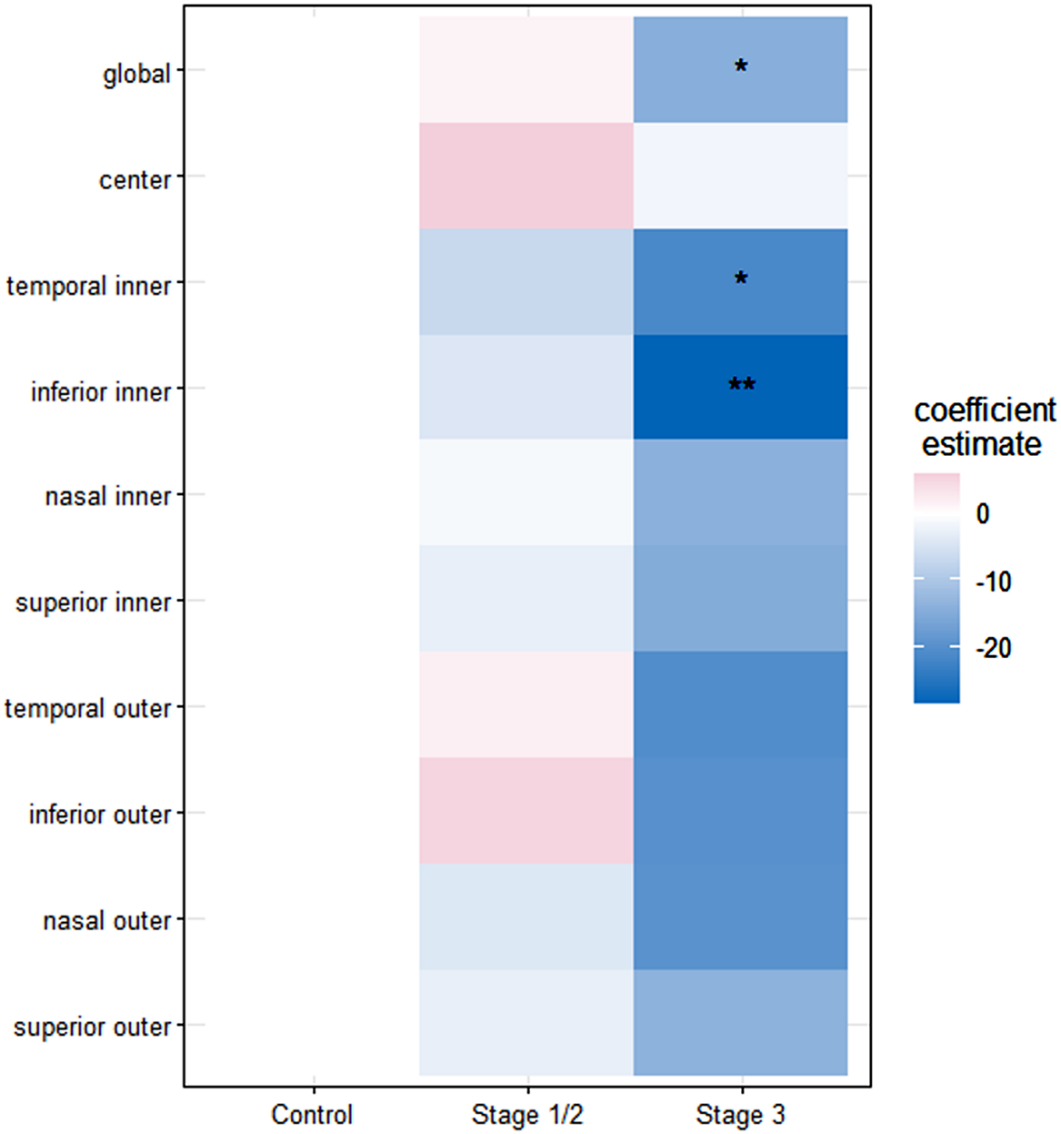
Graphical representation of linear-mixed model results from the subgroup analysis, for example, macular telangiectasia (MacTel) eyes without any manifest ellipsoid zone (EZ) loss, for the global (*first row*) and the topographic (*second to final row*) assessment of the relative ellipsoid zone reflectivity (rEZR) and its association with MacTel disease staging. The extent of rEZR differences between MacTel stages and healthy controls is indicated by the color-coded (from *light red to dark blue*) coefficient estimates. Coefficient estimates of higher negative values, indicating more pronounced rEZR decreases of MacTel eyes compared to controls, are represented by *darker blue colors*. *P* values (*** = *P* value < 0.001; ** = *P* value <0.01; * *P* value <0.05). Eyes with MacTel disease stages 4/5 were not included in this analysis, as all of them did show manifest EZ loss and did hence not fit into the analyzed subgroup.

For detailed results of the topographic rEZR analysis in MacTel eyes (without manifest EZ loss) of different disease stages, please see Table 5. Figure 5 demonstrates a graphical representation of rEZR results in MacTel eyes without manifest EZ loss for both global and topographical assessment.

**Table 5.** Linear-Mixed Model Relative Ellipsoid Zone Reflectivity (rEZR) Results of all Assessed Macular Telangiectasia Type 2 (MacTel) Eyes Without Manifest Ellipsoid Zone (EZ) Loss, Considering Topographic Distribution and Disease Staging, Compared to Controls (Reference)

	Disease Stage	Coefficient Estimates [AU]	95% Confidence Interval [AU]	*P* Value
Global	Intercept	105.8	83.2 to 128.6	
	1/2	1.4	−11.6 to 14.4	0.829
	3	−14.2	−28.4 to −0.04	0.049
Center	Intercept	44.3	24.4 to 64.2	
	1/2	5.9	−5.4 to 17.2	0.305
	3	−1.7	−14.0 to 10.7	0.793
Temporal inner	Intercept	112.0	83.9 to 140.1	
	1/2	−6.6	−23.5 to 10.2	0.441
	3	−21.2	−39.3 to −3.2	0.021
Inferior inner	Intercept	120.9	89.1 to 152.8	
	1/2	−4.3	−23.1 to 14.6	0.656
	3	−28.4	−48.7 to −8.1	0.006
Nasal inner	Intercept	95.9	63.5 to 128.3	
	1/2	−1	−20.2 to 18.2	0.919
	3	−13.8	−34.2 to 6.6	0.185
Superior inner	Intercept	125.2	90.6 to 159.8	
	1/2	−2.8	−22.7 to 17.0	0.78
	3	−14.8	−37.0 to 7.3	0.19
Temporal outer	Intercept	136.5	102.8 to 170.1	
	1/2	2.1	−18.0 to 22.1	0.84
	3	−20.4	−42.1 to 1.3	0.066
Inferior outer	Intercept	134	102.2 to 165.7	
	1/2	5.2	−13.5 to 23.9	0.585
	3	−19.7	−39.8 to 0.4	0.055
Nasal outer	Intercept	146.6	107.6 to 185.6	
	1/2	−4	−27.3 to 19.2	0.733
	3	−19.5	−44.2 to 5.2	0.121
Superior outer	Intercept	128.2	90.9 to 165.4	
	1/2	−2.8	−24.3 to 18.7	0.799
	3	−13.7	−37.5 to 10.1	0.26

## Discussion

This is the first study evaluating the rEZR, a potential novel biomarker for outer retinal integrity, in patients with MacTel, demonstrating lower rEZR values in MacTel eyes compared to controls. This difference was revealed to underlie a spatial dependence with pronounced differences in the predilection area of MacTel which, moreover, were also apparent in eyes without manifest EZ loss. Additionally, the rEZR was shown to be associated with disease severity indicated by lower rEZR values in advanced MacTel stages.

Given current assumptions of photoreceptors’ mitochondria and their junctional complexes with Müller cells being the source of the EZ and ELM reflectivity signal in SD-OCT imaging, respectively, their assessment has become a well-established indicator for photoreceptors’ integrity and outer retina degeneration in MacTel.^24^^,^^25^ Whereas a qualitative, structural loss of EZ and ELM indicates not only fulminant and progressive, but also and more importantly irreversible damage of the outer retina, a quantification of the EZ and ELM reflectivity signal in SD-OCT imaging might be helpful here to assess earlier outer retinal changes preceding and/or occurring beyond manifest EZ and ELM loss.

This study demonstrates in the topographical assessment that across all included MacTel eyes the rEZR decrease was most and significantly pronounced in the temporal inner and inferior inner ETDRS subfield with a CE of −23.2 (−35.7 and −10.8) AU and of −25.2 (−39.4 and −11.1) AU (both: *P* < 0.001). In the global analysis, however, the rEZR decrease in MacTel eyes was revealed to be both less pronounced (CE of −7.7 [−17.8 and 2.5] AU) and not statistically significant (*P* = 0.141). These findings are in line with previous studies highlighting the topographical predilection of retinal alterations in MacTel being mostly located temporal and inferior to the fovea, in the so-called “MacTel area.”^2^^,^^26^^–^^28^ Barthelmes et al. have previously identified an EZ reflectivity reduction in MacTel eyes, which was also spatially confined to the temporal-inferior region of the parafovea.^17^ Given the chosen study design by Barthelmes and colleagues, including the small sample size of 14 MacTel eyes, the use of a different (and no longer available) OCT device as well as having not normalized the EZ reflectivity (and thus not avoiding potential localized confounders on the EZ signal), their results need to be interpreted very cautiously. But nevertheless, the findings by Barthelmes et al. and our here presented findings indicate underlying photoreceptor/outer retina affection in the perifoveal region, notable also in eyes without manifest EZ loss, which is topographically in line with the current understanding of MacTel pathophysiology.^17^

Besides the topographical aspect, one further important finding of this study is the rEZR's association with MacTel disease severity. In the global and the topographical analysis, the rEZR was shown to decrease with advancing disease stages of MacTel (see Table 3, Fig. 4). Interestingly, we found the inter eye correlation to be reduced in eyes of more advanced disease stages. Although distinct MacTel-associated structural alterations have been identified in SD-OCT imaging, only limited data are available on their precise association with disease staging according to Gass and Blodi.^29^^–^^31^ The here presented study, however, shows an association of the rEZR with the CFP-based disease staging, which again highlights underlying and in SD-OCT imaging detectable photoreceptor and outer retina alterations present already in early disease stages and which increase given the chronic-progressive nature of MacTel.

Several limitations need to be considered in the interpretation of this study. Although this is the first study evaluating the rEZR in MacTel, other concurrent SD-OCT based features of MacTel have not been considered here. Second, this study aimed in only assessing structural image data of patients with MacTel and does therefore not include any analysis of visual function and its correlation with the rEZR. Further, because analyses were performed on a cross-sectional image data set, further longitudinal studies are needed to evaluate the rEZR's prognostic value for disease progression. However, a strength of this study is the prospectively acquired image data set in context of an international multicenter natural history study (www.mactelresearch.org) warranting therefore high quality and comparability. Additionally, it needs to be mentioned that the rEZR was determined as the ratio of the EZ to ELM reflectivity (see Methods section for further details). As explained, the ELM is assumed to represent junctional complexes between Müller cells and photoreceptors, which can both alter during the disease process of MacTel.^32^ To date, histopathologic analysis of Müller Cells in MacTel has only been performed in a small number of eyes, large scale analyses are lacking.^4^ But whereas ELM alterations on high resolution retinal imaging have been described predominantly in areas of manifest EZ-loss, their impact on the present analysis is here assumed to be negligible as EZ loss areas were systematically excluded from any analysis.^25^^,^^33^^–^^35^

In conclusion, this is the first study assessing the rEZR in MacTel revealing a decrease of the rEZR being associated with disease staging and which was spatially more pronounced in the parafoveal predilection area of MacTel. These findings are in line with the current understanding of the MacTel pathophysiology supporting the rEZR to be a potential innovative SD-OCT biomarker for outer retinal integrity and MacTel severity. This study warrants further analyses, including a refined structure-function-correlation and evaluation of the rEZR's prognostic value for disease progression in MacTel.

## References

[1] Heeren TFC, Holz FG, Issa PC. First symptoms and their age of onset in macular telangiectasia type 2. *Retina*. 2014; 34(5): 916–919. 24351446 10.1097/IAE.0000000000000082

[2] Charbel Issa P, Gillies MC, Chew EY, et al. Macular telangiectasia type 2. *Prog Retin Eye Res*. 2013; 34: 49–77. 23219692 10.1016/j.preteyeres.2012.11.002 PMC3638089

[3] Peto T, Heeren TFC, Clemons TE, et al. Correlation of clinical and structural progression with visual acuity loss in macular telangiectasia type 2. *Retina*. 2018; 38(1): S8–S13. 28505012 10.1097/IAE.0000000000001697 PMC8326288

[4] Powner MB, Gillies MC, Zhu M, Vevis K, Hunyor AP, Fruttiger M. Loss of Müller's cells and photoreceptors in macular telangiectasia type 2. *Ophthalmology*. 2013; 120(11): 2344–2352. 23769334 10.1016/j.ophtha.2013.04.013

[5] Lin GG, Scott JG. Müller glia cell reprogramming and retina regeneration. *Nat Rev Neurosci*. 2012; 100(2): 130–134. 10.1038/nrn3723 PMC4249724 24894585

[6] Gaudric A, Krivosic V, Tadayoni R. Outer retina capillary invasion and ellipsoid zone loss in macular telangiectasia type 2 imaged by optical coherence tomography angiography. *Retina*. 2015; 35(11): 2300–2306. 26441270 10.1097/IAE.0000000000000799

[7] Sallo FB, Peto T, Egan C, et al. “En face” OCT imaging of the IS/OS junction line in type 2 idiopathic macular telangiectasia. *Investig Ophthalmol Vis Sci*. 2012; 53(10): 6145–6152. 22899757 10.1167/iovs.12-10580 PMC4608676

[8] Sallo FB, Leung I, Mathenge W, et al. The prevalence of type 2 idiopathic macular telangiectasia in two African populations. *Ophthalmic Epidemiol*. 2012; 19(4): 185–189. 22364548 10.3109/09286586.2011.638744

[9] Pauleikhoff D, Bonelli R, Dubis AM, et al. Progression characteristics of ellipsoid zone loss in macular telangiectasia type 2. *Acta Ophthalmol*. 2019; 97(7): e998–e1005. 30968592 10.1111/aos.14110 PMC6785352

[10] Heeren TFC, Kitka D, Florea D, et al. Longitudinal correlation of ellipsoid zone loss and functional loss in macular telangiectasia type 2. *Retina*. 2018; 38(Suppl 1): S20–S26. 28541959 10.1097/IAE.0000000000001715 PMC8326028

[11] Chew EY, Clemons TE, Peto T, et al. Ciliary neurotrophic factor for macular telangiectasia type 2: results from a phase 1 safety trial. *Am J Ophthalmol*. 2015; 159(4): 659–666.e1. 25528956 10.1016/j.ajo.2014.12.013 PMC4361328

[12] Staurenghi G, Sadda S, Chakravarthy U, Spaide RF. Proposed lexicon for anatomic landmarks in normal posterior segment spectral-domain optical coherence tomography: the IN•OCT consensus. *Ophthalmology*. 2014; 121(8): 1572–1578. 24755005 10.1016/j.ophtha.2014.02.023

[13] Hoang QV, Linsenmeier RA, Chung CK, Curcio CA. Photoreceptor inner segments in monkey and human retina: mitochondrial density, optics, and regional variation. *Vis Neurosci*. 2002; 19(4): 395–407. 12511073 10.1017/s0952523802194028

[14] Litts KM, Okada M, Heeren TFC, et al. Longitudinal assessment of remnant foveal cone structure in a case series of early macular telangiectasia type 2. *Transl Vis Sci Technol*. 2020; 9(4): 1–12. 10.1167/tvst.9.4.27 PMC7396184 32818114

[15] Gin TJ, Wu Z, Chew SKH, Guymer RH, Luu CD. Quantitative analysis of the ellipsoid zone intensity in phenotypic variations of intermediate age-related macular degeneration. *Investig Ophthalmol Vis Sci*. 2017; 58(4): 2079–2086. 28388704 10.1167/iovs.16-20105

[16] Borrelli E, Sacconi R, Zuccaro B, et al. Photoreceptor alteration in intermediate age-related macular degeneration. *Sci Rep*. 2020; 10(1): 1–9. 33273666 10.1038/s41598-020-78201-9 PMC7713116

[17] Barthelmes D, Gillies MC, Sutter FKP. Quantitative OCT analysis of idiopathic perifoveal telangiectasia. *Investig Ophthalmol Vis Sci*. 2008; 49(5): 2156–2162. 18436849 10.1167/iovs.07-0478

[18] Gass JDM, Blodi BA. Idiopathic juxtafoveolar retinal telangiectasis: update of classification and follow-up study. *Ophthalmology*. 1993; 100(10): 1536–1546. 8414413

[19] Thiele S, Isselmann B, Pfau M, et al. Validation of an automated quantification of relative ellipsoid zone reflectivity on spectral domain-optical coherence tomography images. *Transl Vis Sci Technol*. 2020; 9(11): 1–10. 10.1167/tvst.9.11.17 PMC7581490 33133775

[20] Wu Z, Ayton LN, Guymer RH, Luu CD. Relationship between the second reflective band on optical coherence tomography and multifocal electroretinography in age-related macular degeneration. *Investig Ophthalmol Vis Sci*. 2013; 54(4): 2800–2806. 23532524 10.1167/iovs.13-11613

[21] Powner MB, Gillies MC, Tretiach M, et al. Perifoveal Müller cell depletion in a case of macular telangiectasia type 2. *Ophthalmology*. 2010; 117(12): 2407–2416. 10.1016/j.ophtha.2010.04.001 PMC2974049 20678804

[22] [No authors listed]. Photocoagulation for diabetic macular edema: early treatment diabetic retinopathy study report number 1 early treatment diabetic retinopathy study research group. *Arch Ophthalmol*. 1985; 103(12): 1796–1806. 2866759

[23] Diedenhofen B, Musch J. Cocor: a comprehensive solution for the statistical comparison of correlations. *PLoS One*. 2015; 10(4): 1–12. 10.1371/journal.pone.0121945 PMC4383486 25835001

[24] Chew EY, Clemons TE, Jaffe GJ, et al. Effect of ciliary neurotrophic factor on retinal neurodegeneration in patients with macular telangiectasia type 2: a randomized clinical trial. *Ophthalmology*. 2019; 126(4): 540–549. 30292541 10.1016/j.ophtha.2018.09.041 PMC8365464

[25] Ledolter AA, Ristl R, Palmowski-Wolfe AM, et al. Macular telangiectasia type 2: multimodal assessment of retinal function and microstructure. *Acta Ophthalmol*. 2021. 10.1111/aos.15072 34854225

[26] Runkle AP, Kaiser PK, Srivastava SK, Schachat AP, Reese JL, Ehlers JP. OCT angiography and ellipsoid zone mapping of macular telangiectasia type 2 from the AVATAR study. *Investig Ophthalmol Vis Sci*. 2017; 58(9): 3683–3689. 28727884 10.1167/iovs.16-20976 PMC5518977

[27] Sauer L, Vitale AS, Modersitzki NK, Bernstein PS. Longitudinal fluorescence lifetime imaging ophthalmoscopy analysis in patients with macular telangiectasia type 2 (MacTel). *Retina*. 2021; 41(7): 1416–1427. 34137386 10.1097/IAE.0000000000003055

[28] Solberg Y, Dysli C, Wolf S, Zinkernagel MS. Fluorescence lifetime patterns in macular telangiectasia type 2. *Retina*. 2019; 40(99): 1. 10.1097/IAE.0000000000002411 PMC6924947 30664123

[29] Yannuzzi LA, Bardal AMC, Freund KB, Chen KJ, Eandi CM, Blodi B. Idiopathic macular telangiectasia. *Arch Ophthalmol*. 2006; 124(4): 450–460. 16606869 10.1001/archopht.124.4.450

[30] Gaudric A, Ducos De Lahitte G, Cohen SY, Massin P, Haouchine B. Optical coherence tomography in group 2A idiopathic juxtafoveolar retinal telangiectasis. *Arch Ophthalmol*. 2006; 124(10): 1410–1419. 17030708 10.1001/archopht.124.10.1410

[31] Cohen SM, Cohen ML, El-Jabali F, Pautler SE. Re: optical coherence tomography findings in nonproliferative group 2a idiopathic juxtafoveal retinal telangiectasis. *Retina*. 2007; 27(2): 59–66. 17218917 10.1097/01.iae.0000256663.94734.e1

[32] Spaide RF, Curcio CA. Anatomical correlates to the bands seen in the outer retina by optical coherence tomography: literature review and model. *Retina*. 2011; 31(8): 1609–1619. 21844839 10.1097/IAE.0b013e3182247535 PMC3619110

[33] Litts KM, Zhang Y, Bailey Freund K, Curcio CA. Optical coherence tomography and histology of age-related macular degeneration support mitochondria as reflectivity sources. *Retina*. 2018; 38(3): 445–461. 29210936 10.1097/IAE.0000000000001946 PMC6230433

[34] Kim YH, Chung YR, Oh J, et al. Optical coherence tomographic features of macular telangiectasia type 2: korean Macular Telangiectasia Type 2 Study—Report No. 1. *Sci Rep*. 2020; 10(1): 1–9. 33024250 10.1038/s41598-020-73803-9 PMC7538897

[35] Wang Q, Tuten WS, Lujan BJ, et al. Adaptive optics microperimetry and OCT images show preserved function and recovery of cone visibility in macular telangiectasia type 2 retinal lesions. Invest Ophthalmol Vis Sci. 2015;56(2):778-786. 10.1167/iovs.14-15576 PMC4315435 25587056

